# One Hundred Consecutive Cases of Skin Disease, III

**Published:** 1891-03

**Authors:** M. B. Hutchins

**Affiliations:** Lecturer on Diseases of the Skin, Atlanta Medical College


### ONE HUNDRED CONSECUTIVE CASES OF SKIN DISEASE.

III

BY M. B. HUTCHINS, M. D.,
Lecturer on Diseases of the Skin, Atlanta Medical College.

[NOTE.—In preceding article, on page 726, in directions for dose, following prescription in middle of page, 1 oz. should be 1 drachm.

Eczema of scrotum, two cases. First, that of a druggist, aged forty-two. Left half, and posterior surface of scrotum and anterior portion of perineum affected. Thickening marked, and there were deep fissures, many the result of scratching, for the severe itching which was present. Patient, however, complained more of "burning" than of itching. There was some "oozing" from the fissures. Here and there were a few large papules, excoriated and slightly discharging.

On the scalp there were a few sero-crusted or excoriated, discrete lesions, itchy and aggravated by scratching. Sensations, at times, of dizziness and weakness.

A " pigment" composed of—

℞. Olei. cadenii, ʒii.

Ether, sulph.,

Sp. vini. rect., aa ℥ii.

M.

was applied to scrotum and perineum, but found ineffective. Then the "*Salicylic*, *zinc* and *diachylon ointment*," as already described, was ordered applied on cloth, and held by suspensory bandage during the day. The ointment was to be frequently rubbed on scrotum and perineum at night, especially if itching recurred.

Within four days the fissures were healed, skin was quite soft and smooth, but now more itching and less " burning." (The " burning " was in the fissures.) There was no relapse, but the patient excoriated the scrotum several times by scratching. I may here say that itching is the most prominent and persistent subjective symptom of eczema. In a month the patient was well enough to return to his home in Florida. The treatment of the scalp was as will be given under another head, but was effectually counterbalanced by the industrious use which the patient made of his nails.

Second of scrotum; gentleman of thirty-five, also from Florida. Skin but slightly thickened and presenting a faint "glazed" appearance. Symptoms chiefly subjective, itching being severe, especially at night. (At base of penis and on right buttock irregular, reddish segments of circles, suggestive of " ringworm." Not treated.) Ointment, as in case one, did not relieve itching, and the patient was not seen again, having returned to Florida at the time he began treatment. Ointments seemed to have been improperly made, at first, and finally he seemed to find nothing satisfactory, and probably is still itching.

Eczema of the foot, one case, car inspector, aged thirty-four. Present five months despite all treatment. On back of the small toes of the left foot, over their base and the anterior half of skin covering metatarsal bones, sharply defined above, thickening, slight redness, scaling and here and there a denuded point from which oozing occurred. Itching present at times. Bromidrosis also present. Disease had been aggravated by bathing in salt water.

℞. Ac. salicyl., gr. xv.

Magnesiæ carb., gr. x.

Ungt. zn. ox., ℥i.

M.

Sig.—Apply on cloth and bind in place.

This produced immediate improvement, and after all appearance of "activity" in disease subsided, pix. liquida ʒi was substituted for magnesia carbonate. Itching, which had been very persistent, was relieved by the addition to the ointment of ʒii spir. camphoræ. Patient's foot was entirely well in a month.

Of "general" eczema, or that occupying the greater part of the skin surface, first case that of a married woman, aged twenty-two. Disease began on hands about four weeks previously as papules and vesicles, and when seen almost the entire skin surface was covered with pale red papules and vesicles. Here and there was a pustule. On wrist and back of right hand the skin had become excoriated, and there was quite profuse discharge and some crusting. Hands doubtless made worse from use of strong soap. There was oozing from skin of left auditory meatus and slight crusting. This case was a clear example of the papulo-vesicular type of eczema.

The symptoms were suggestive of local irritation, as in what is generally known as "poisoning" from "poison ivy" (rhus toxicodendron) or the poison from sumach, but there was no history of exposure to any external irritation of this character. Patient was four months pregnant, fifth child, and there was a question as to the influence of this condition.

℞. Acid salicyl., ʒii.

Zinci. oxidi.,

Amyli, aa ℥i.

Ungt. simp., ad. ℥viii.

M.

Sig.—Keep diseased surface covered with this and protect hands with rubber gloves.

There was steady improvement, and the patient was practically well in twelve days, after which I did not see her.

A second case, general in localization, was that of an old lady, aged eighty-five. Type squamous, chronic; duration five months. Began on body and about axillæ as "weeping" and crusting patches, soon becoming thickened and very scaly. When seen, on the back of the neck, the axillæ and adjacent skin, various parts of the body, arms, legs and about pudenda and buttocks were thickened patches of glazed appearance, upon which large, thin scales formed. Here and there variously sized fissures were seen. General skin atrophic (senile) and itchy.

℞. Picis liq., ʒiv.

Camphoræ, ʒii.

Ungt. diachyli, ℥iv.

M.

Sig.—Test on one patch, if acts well apply to all, keeping on constantly.

When the ointment was, finally, properly applied there was marked improvement. After three weeks—

℞. Cascar. sag. F.E., ℥ss.
Tinct. nucis. vom., ʒi.
Tinct. cinchon. comp., ℥ii.
Aquæ, ad. ℥iv.

M.

Teaspoonful after meals was given for "tonic" effect and to keep bowels open.

℞. Saponis viridis, ℥ii.
Sp. vini. rect., ℥i.

M.

was used to "scrub" patches once daily, after which the ointment was reapplied. The pix liquida was decreased one-half, and acid salicylic grs. 10 to the ounce of ointment was added about six weeks from beginning of treatment. Despite the age of the patient and the difficulty of getting the treatment thoroughly applied, the disease became much less troublesome, and so far as I learned, remained so until the patient's death, four months later. A relative says disease first became severe after a course of *royal germateur*.

A curious case of "*trade eczema*" was that of a negro hostler, aged twenty-two. Duration four days. On, and between fingers and forearms, on rest of skin surface slightly, numerous acuminate, pin-head sized papules. "Special arrangement" or localization, not present. Some of the papules pierced by a hair. No vesicles visible even with a lens. Only sensation was that of slight pricking when warm. Allowed the dust from the horses curried to remain on skin, though he said he washed his hands and wrists. This dust was probably partly composed of dried excrement, from the stalls, and irritated the skin. No evidence of syphilis or scabies.

Ordered free use of soap and water, a nightly bath and the application of the "pigment" of *pitch*, *ether and alcohol.* Perhaps so much soap and water was against his scruples, as he did not return.

The case of erythematous eczema in the child, described in Article I., is an example of general eczema of this type, viz.: erythematous. Hence, we have described three types of general eczema, the erythematous, the papulo-vesicular, and the chronic squamous. As a general rule an eczema does not remain of a distinct type throughout its course, but may represent different types at different times or belong to the "mixed" type. While acute and active, the disease must recieve soothing, a stringent and protective treatment; when chronic and indolent, "stimulating" treatment is, as a rule, indicated, but there should be no effort at destruction of a chronic eczema by *caustics.*

A case of eczema may last, under proper treatment, from three weeks to three months, or longer, but should always be considered curable with proper treatment and the ability to regulate the general health.

(*Seborrhoic Eczema next article.*)

16½ *Whitehall St.*

